# Design and Characterization of 5 μm Pitch InGaAs Photodiodes Using In Situ Doping and Shallow Mesa Architecture for SWIR Sensing †

**DOI:** 10.3390/s23229219

**Published:** 2023-11-16

**Authors:** Jules Tillement, Cyril Cervera, Jacques Baylet, Christophe Jany, François Nardelli, Thomas Di Rito, Sylvain Georges, Gabriel Mugny, Olivier Saxod, Olivier Gravrand, Thierry Baron, François Roy, Frédéric Boeuf

**Affiliations:** 1 STMicroelectronics, 850 Rue Jean Monnet, 38054 Crolles, France; 2 Univ. Grenoble Alpes, CEA, Leti, F38000 Grenoble, France; 3 CNRS LTM, 38054 Grenoble, France

**Keywords:** InGaAs, photodiode, SWIR, shallow-mesa, small pitch

## Abstract

This paper presents the complete design, fabrication, and characterization of a shallow-mesa photodiode for short-wave infra-red (SWIR) sensing. We characterized and demonstrated photodiodes collecting 1.55 μm photons with a pixel pitch as small as 3 μm. For a 5 μm pixel pitch photodiode, we measured the external quantum efficiency reaching as high as 54%. With substrate removal and an ideal anti-reflective coating, we estimated the internal quantum efficiency as achieving 77% at 1.55 μm. The best measured dark current density reached 5 nA/cm^2^ at −0.1 V and at 23 °C. The main contributors responsible for this dark current were investigated through the study of its evolution with temperature. We also highlight the importance of passivation with a perimetric contribution analysis and the correlation between MIS capacitance characterization and dark current performance.

## 1. Introduction

The development of short-wave infra-red (SWIR) sensors is being led by the growing demand in various fields such as security, automotive, industry, and agriculture [1]. In the SWIR range between 1 and 2.5 μm, silicon is quasi transparent ($\lambda_{cut,Si}=1.1\;\mu m$) hence, to absorb an SWIR photon, the absorption material needs to be changed. The main candidates to do so and to be integrated in a SWIR sensor are PbS quantum dots in quantum films [2], In_0.53_Ga_0.47_As [3,4,5,6], Ge on Si [7,8], or graphene [9,10].

**Figure 1:**
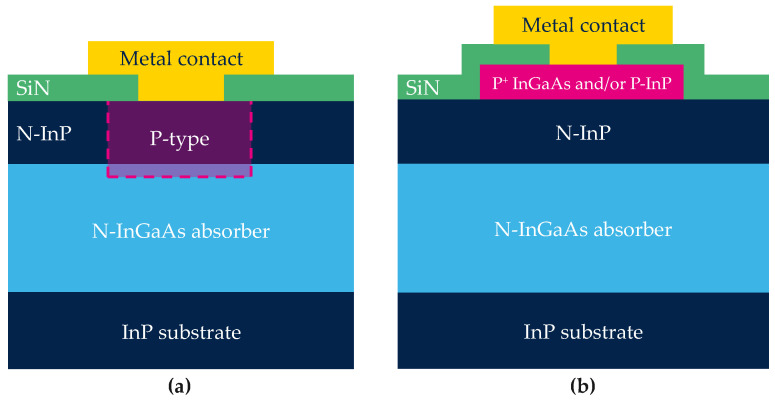
Schematic cross section of the photodiode after different processes. (**a**) Photodiode fabricated by Zn diffusion or Be implantation; (**b**) photodiode fabrication using shallow mesa technique.

In_0.53_Ga_0.47_As is latticed-matched to InP and has a band gap of 0.74 eV at room temperature [11]. This band gap is suitable for SWIR collection, leading to a cut-off wavelength of 1.7 μm. The state-of-the-art for an InGaAs image sensor integrated on a Si-based read-out integrated circuit (ROIC) is a 5 μm pixel pitch with quantum efficiency (QE) greater than 75% at 1.2 μm. The dark current is reported to be as low as 2 nA/cm^2^ at −0.1 V and at 23 °C [3]. The standard structure for an InGaAs-based SWIR sensor is shown in Figure 1a. It is composed of a thick low doped n-InGaAs absorption layer epitaxied on an InP substrate. III-V materials, such as InGaAs, lack high quality native dielectric and are sensitive to electrical surface defect density [12,13]. To minimize surface leakage current at the InGaAs/dielectric interface, the InGaAs layer is covered by an InP layer which has a larger band gap of 1.34 eV at 25 °C [14]. This layer is used as passivation to move the poor electronic interface away from the active region. In such structures where the InGaAs layer is the active region, it is a priority to have the lowest defect density possible at the interfaces and in the bulk.

For the InGaAs photodiode fabrication, the crucial process step is the definition of each pixel by the creation of a *P* region in the *N*-type stack. Literature reports different alternatives for this purpose such as Zn diffusion [3,4,15], Be implantation [16,17], or shallow mesa-type architecture [18,19].

Today, the standard industrial process to create the *P*-type area relies on the Zn diffusion process. First, a SiN dielectric mask is deposited on the *N*-type stack. The dielectric is then opened, and Zn is diffused through this aperture. Zn atoms diffuse in the InP layer and reach the InGaAs absorption layer, creating the *P*-type region. Figure 1a shows the schematic cross section of the photodiode after the diffusion process. To scale down the pixel pitch to a few μm dimensions, the isotropic behaviour of the diffusion presents a challenge. In addition to this isotropic diffusion, it has been shown that Zn diffuses laterally faster in InGaAs than in InP [20]. Greater doping near the interface in the InGaAs layer seems to successfully limit the lateral spread for a 15 μm pixel pitch [20]. With the reduction of the pixel pitch, this diffusion process is harder to control as the distance between the two nearest *P*-type wells reduces. This phenomenon might then limit the final resolution of the image sensor.

Ionic implantation (I/I) could be an interesting solution to overcome the issue of lateral diffusion. I/I is commonly used in Si processes to precisely locate the dopants in the structure. The main drawback of this technique is due to the introduction of many defects. It is mandatory to cure as many defects as possible during the anneal following the implantation to avoid the generation of dark current. Few studies have reported the damage in InP/InGaAs photodiodes caused by I/I [21] but curing defects in the III-V structure have been studied mostly in GaAs [22,23,24,25]. After I/I, the amorphous III-V layer shows poor recrystallization because of the binary or ternary nature of the material. The differences in solubility contribute to the formation of disorder in the lattice [22]. We previously reported the use of Be implantation to define the InGaAs/InP photodiode. The published dark current is at best in the range of the μA/cm^2^ [17].

Finally, combining in situ Zn-doped with photolithography and etching tools to define the *p* region could allow control of the lateral size of the diode. Only a few groups have reported studies of such architecture [18,19,26]. Unlike other studies, our process for mesa-type device fabrication only removes the p-doped region from the top stack. The final schematic structure is presented on Figure 1b and is referred to as shallow-mesa architecture. The stack must be carefully designed to allow the collection of photo-carriers generated in the InGaAs absorption layer (colour: light blue in Figure 1).

For the fabrication of the InGaAs image sensor with pixel pitch below 5 μm, the Cu–Cu hybridization technique from Si image sensors can be adapted to InGaAs image sensors [3] as this method is already in mass production for pixel pitch down to 1 μm [27].

The InGaAs image sensor fabrication targets a Si-CMOS compatible process in large format for high volume. First, epitaxial dies would be bonded on a large format carrier Si wafer. Then, the III-V photodiode process would be performed with up-to-date Si tools. Lastly, the photodiodes would be bonded on the Si ROIC. In this perspective, the in situ doping alternative is a low thermal budget process which is an asset for this integration in a Si-CMOS compatible fab. This kind of Si-CMOS compatible process flow is also presented in [3] with Zn diffused photodiodes.

## 2. Design of the Shallow-Mesa Architecture

**Figure 2:**
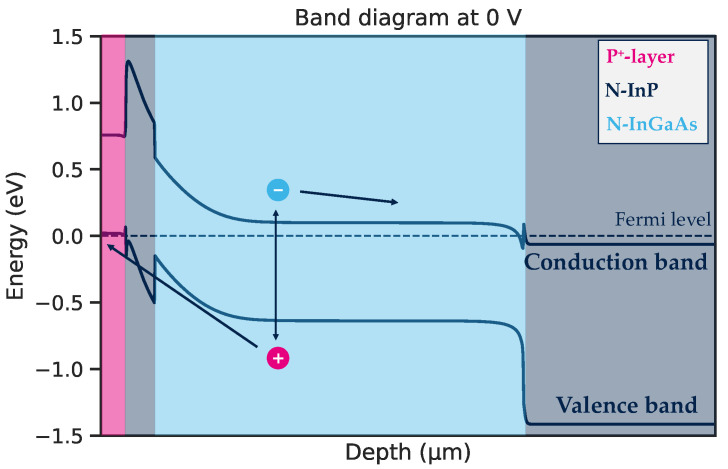
Band diagram of simulated structure at equilibrium with the photogenerated pair schematically represented with their path of collection.

The goal of the designed structure is to collect the photogenerated carriers from the InGaAs absorption layer at low reverse bias, to match the ROIC needs. The minority carriers must go through the heterojunction visible on the simulated band diagram presented in Figure 2. The *N*-InP layer passivates the small gap InGaAs layer as mentioned previously, but the heterojunction introduces an unfavourable barrier for the hole collection. To overcome this barrier, the structure is designed to intrinsically introduce an electric field that attracts carriers at equilibrium.

The device simulation is performed with Synopsys Sentaurus software [28] and aims at finding a set of parameters which suppresses the electrostatic barrier for the collection path. The simulation is conducted considering Auger and radiative recombination as well as Shockley–Read-Hall (SRH) recombination. Intrinsic and quality related material parameters are based on the literature [29,30] and are used as simulation inputs for model calibration.

**Figure 3:**
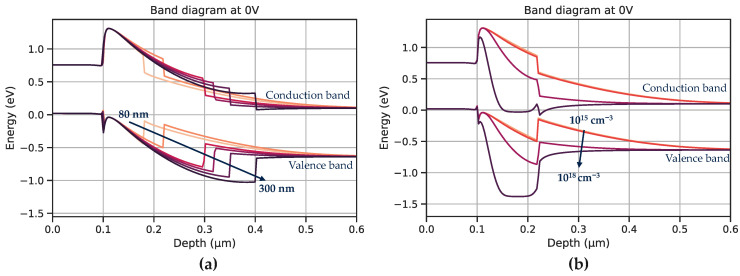
Top zoom of the structure—Impact of the *N*-InP (**a**) thickness and (**b**) doping on the band diagram at equilibrium.

For the carrier collection, the electric field can be modulated by three key parameters which are the doping and thickness of the barrier *N*-InP layer and the doping concentration of the *P*-type contact layer. Figure 3 shows the evolution of the band diagram at equilibrium for a fixed *P*-type layer doping. The impact of the *N*-InP thickness ranging from 80 nm to 300 nm is presented Figure 3a where the thicker the *N*-InP, the greater the electrostatic barrier. This is also visible in Figure 3b as the larger the doping of the *N*-InP layer, the greater the barrier (doping values between 10^15^ and 10^18^ cm^−3^).

**Figure 4:**
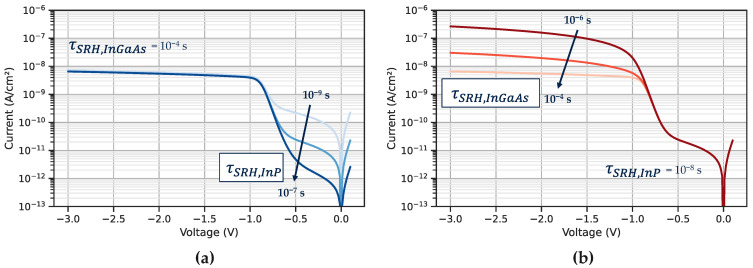
Simulated dark current with TCAD Synopsys tools [28]. (**a**) Shows evolution of the dark current when the InP SRH lifetime is modulated; (**b**) evolution of the dark current when the InGaAs SRH lifetime is modulated.

The simulation helps us to separate the contribution of dark current from InP and from the InGaAs layer. The origin of each dark current was verified by changing the carrier lifetime in each material and analysing the evolution on the total dark current. For a non-optimized structure as presented in Figure 4, the simulated dark current shows that carriers collected at low reverse bias (<1 V) are generated in InP (Figure 4a). At higher reverse bias (>1 V), the current of interest is collected in the InGaAs layer (Figure 4b). Our goal was to find a configuration that allows the collection of the InGaAs carriers at low reverse bias.

**Figure 5:**
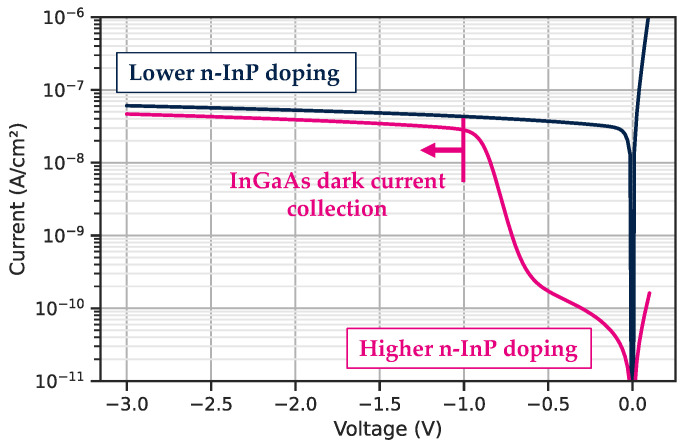
Impact of the doping concentration of the InP barrier on the carrier collection.

The dark blue curve in Figure 5 shows a well-designed structure where the current from InGaAs is collected at quasi null voltage. If the *N*-InP barrier layer is doped too much (in Figure 5 in pink), it increases the electrostatic barrier and blocks the carrier at low bias.

On the other hand, if the *N*-InP cap is too thin to minimize the electrostatic barrier, process constraints appear during the *P*-layer etching process that make it difficult to control uniformity on the wafer.

This theoretical study allows us to find a set of doping concentration and thickness parameters that suppresses the barrier for hole collection. This set of parameters has been used to define the epitaxial stack for photodiode fabrication.

## 3. Fabrication

**Figure 6:**
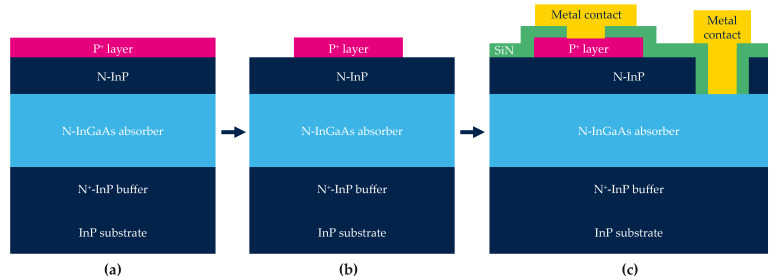
Simplified and schematic process flow of the shallow mesa-type process. (**a**) The full stack; (**b**) the definition of the pixel by etching the *P* layer and (**c**) the encapsulation and fabrication of contacts.

The schematic and simplified process of the shallow-mesa type photodiode is represented in Figure 6. All layers are grown on a 3 inches InP substrate (see Figure 6a).

**Figure 7:**
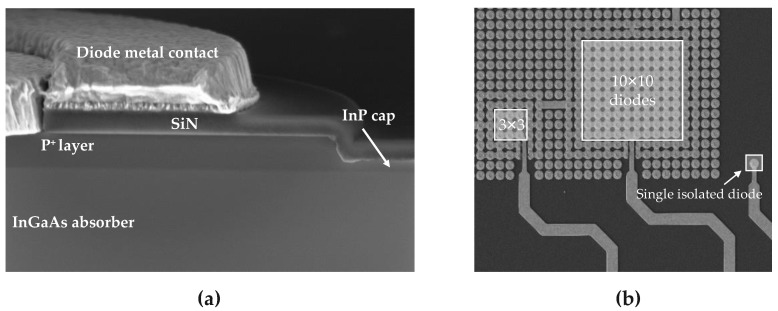
SEM views after the whole process. (**a**) A cross-section of the top stack where the *P* layer is etched and (**b**) a top view of the different configuration of the test structures (single in-array diode is not shown on this SEM view).

The pixel definition is performed by etching the *P*-type layer (see Figure 6b and SEM cross-section view Figure 7a). Then, the device is passivated by a dielectric deposition. The final step is the fabrication of both diode and *N* metal contacts (see Figure 6c).

A specific test structure is designed to emulate a matrix-like environment. Multiple diodes in-array are connected, while two additional rows of diodes at the periphery of this array are independently connected and can be polarized independently. The tested structures shown in Figure 7b could be single isolated or in-array diodes and bundles of three-by-three or ten-by-ten diode bundles in a matrix-like environment.

## 4. Results

The main figure of merit of a sensor is its signal to noise ratio (SNR). It is defined as the ratio of the useful signal divided by its noise. It must be maximized to reach the best performances. It implies high QE for a high signal and low noise. The noise is ultimately limited by the Schottky noise $\sqrt{2qI}$, with I the total current, i.e., the sum of the photonic and the dark current and q the elementary electric charge. For photodiode fabrication, the dark current is a parasitic current and must be negligeable compared to the photonic current.

In the following part, we first compare two processes on 15 μm large pixels to analyse the impact of the passivation on dark current performances. Then, we fabricate 5 μm pixel pitch photodiodes with the most suitable process and characterize it. Lastly, we present 3 μm pixel pitch photodiodes as a demonstration of pixel pitch reduction.

### 4.1. Dark Current Contributor Investigation

**Figure 8:**
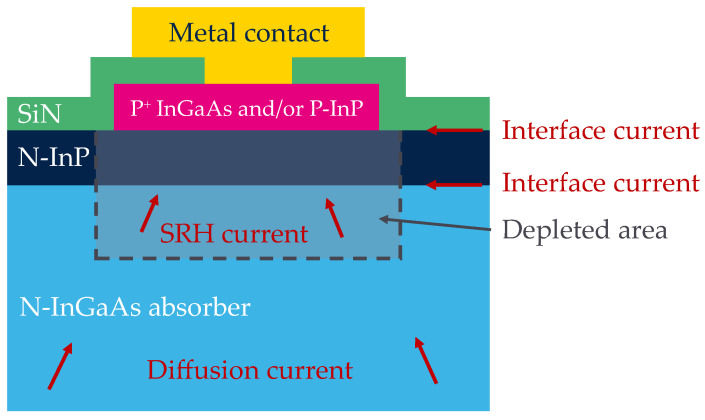
Schematic cross section of the structure with its potential sources of the dark current. Inspired from [32].

The structure is recalled in Figure 8 with the potential sources of dark current. The main sources considered are the diffusion current $J_{diff}\propto \exp\;\left(-\frac{E_{g}}{kT}\right)$ and generation–recombination (GR) current $J_{depl}\propto \exp\;\left(-\frac{E_{g}}{2kT}\right)$ in the depletion region as well as interface current between dielectric/InP and InP/InGaAs. The diffusion current and Shockey–Read–Hall (SRH) generation–recombination (GR) current from the InP layer is neglected as the volume is very small and the band gap is approximately twice the band gap of InGaAs (E_G, InP_ = 1.34 eV and E_G, InGaAs_ = 0.74 eV) [31,32,33,34].

**Figure 9:**
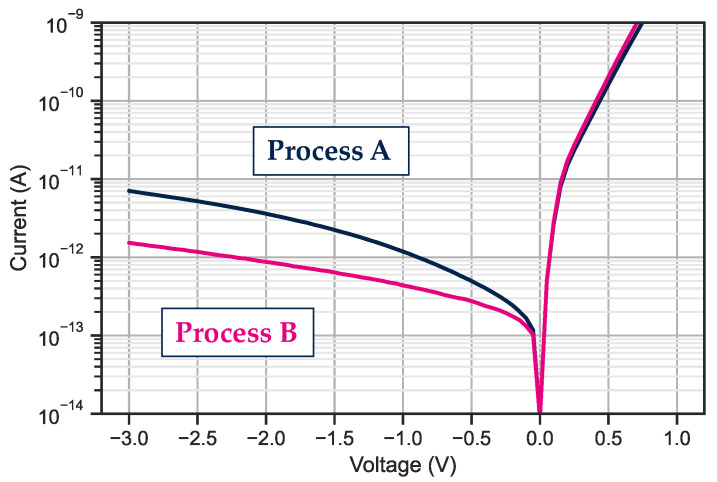
Dark current measurement on 15 μm pitch in a matrix like environment. The curve is the median of more than 100 single in-array diodes measured.

Two processes are compared here to identify the impact of the dielectric for the realization of small pitch photodiode with the lowest dark current possible. The active stack is identical, but the passivation steps are different in terms of dielectric nature. Dark current measurements on 15 μm pixel pitch photodiodes are presented in Figure 9. The evolution is different with the reverse bias. At −0.5 V and at room temperature, the dark current for process A reaches $5\cdot 10^{-13}$ A and for process B, it reaches $2.8\cdot 10^{-13}$ A.

**Figure 10:**
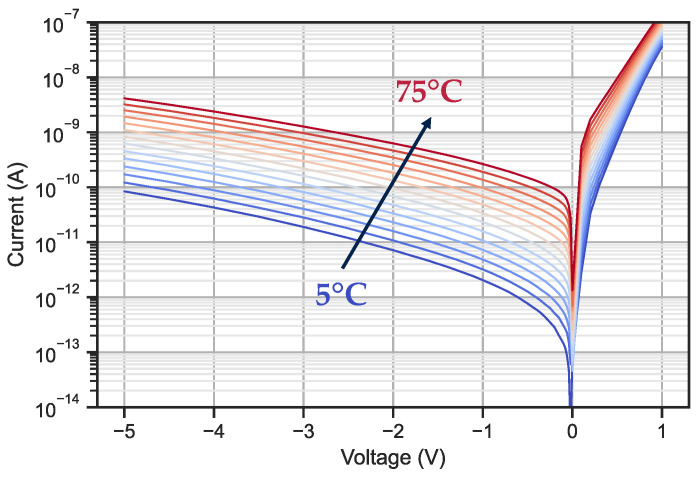
Dark current measurement of the ten-by-ten diode bundle. This measurement is from process B.

The main contributors to the dark current can be studied by analysing its evolution with temperature. We briefly review the theory of the dark current’s dependence with temperature. The generation–recombination current due to SRH processes in the depleted region follows a specific temperature dependence $J_{depl}\propto T^{3/2}\exp\;(E_{G,InGaAs}/2kT)$. If the dark current is limited by the diffusion, the current is proportional to $J_{diff}\propto T^{3}\exp\;(E_{G,InGaAs}/kT)$ [35]. We measured the dark current for temperatures ranging from 5 °C to 75 °C every 5 °C and an example of our results is presented in Figure 10.

**Figure 11:**
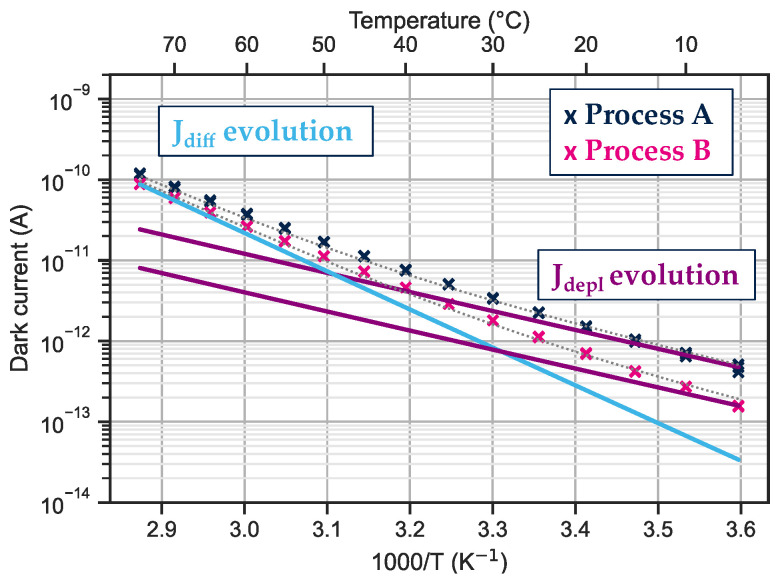
Evolution of the dark current with temperature at −0.1 V. The solid lines show the theoretical evolution of the current limited by diffusion (light blue line) and by generation recombination (purple line). The temperature measurement is performed on a bundle of ten-by-ten 5 μm pixel pitch diodes.

The evolution of the dark current at −0.1 V with temperature is shown in Figure 11. The solid lines show the temperature dependence of the current for diffusion-limited current (light blue) or SRH generation in the depletion current (purple) in the InGaAs absorption layer.

At room temperature and below, the main dark current contributor is the generation–recombination from the depletion region. For temperatures greater than 50 °C, the dark current is mainly limited by the diffusion phenomenon.

For the two processes studied here, the diffusion contribution is the same, but the current from the depletion region is higher for process A than for process B. As the structures and processes are identical except for the dielectric, this difference in J_depl_ indicates that the dielectric has an impact on the dark current.

To further investigate the impact of the dielectric on the dark current, the comparison is conducted on diodes of different sizes ranging from 10 μm to 120 μm to characterize contributions from the bulk ($J_{b}$) and from the periphery ($J_{p}$) based on the following equation:

(1) $$I_{total}=A\times J_{b}+P\times J_{P}$$

**Figure 12:**
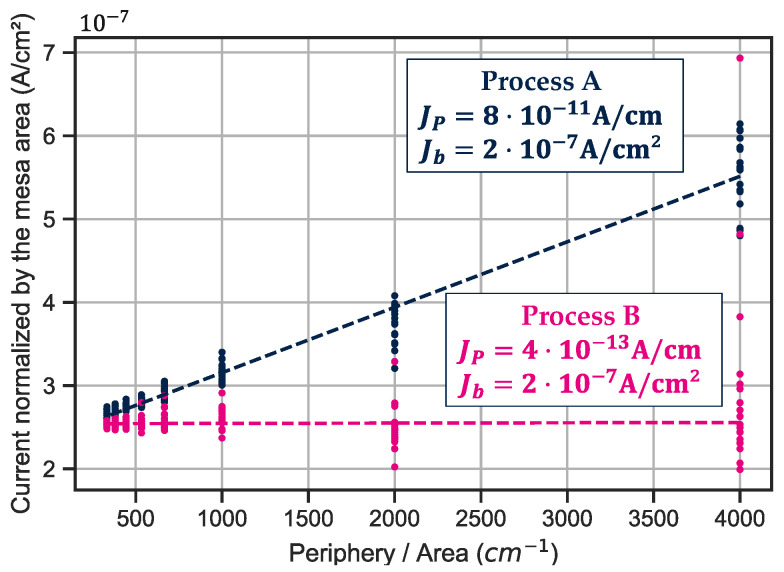
Perimetric and bulk contribution to the global dark current from measurements performed on diodes with diameter ranging from 10 to 120 μm.

A is the area of the diode defined by its *P*-layer diameter and P is the perimeter of the diode defined by the perimeter of the *P*-layer. The measured photodiodes have different *P*/*A* ratios, which were used to plot *I*/*A* as a function of *P*/*A*. By performing a simple linear regression, we were able to distinguish between the bulk and perimeter current densities, $J_{b}$ and $J_{p}$, respectively. In our case, Figure 12 shows the significant different value in $J_{p}$ for the two cases at −0.5 V. As the diode diameter is reduced, the contribution from the perimeter becomes increasingly important relative to the bulk one.

Here, the bulk contribution is even for the two processes as $J_{b}=2\cdot 10^{-7}A/{cm}^{2}$ in both cases; for the perimetric one, in process A, $J_{P}=8\cdot 10^{-11}A/cm$ and for process B, $J_{P}=4\cdot 10^{-13}A/cm$. The perimetric contribution is drastically reduced in process B which implies that this process is much more suitable for small pixel pitch fabrication.

The evolution of the dark current with temperature in Figure 11 indicates a different level of J_depl_ that limits the dark current at room temperature. However, the P/A study presented Figure 12, identifies a clear difference between the two processes. Even though the perimetric contribution is always lower than the bulk one, process A shows a larger perimetric contribution compared to process B. This difference must be due to different dielectric properties obtained with these two processes.

Specific capacitive characterizations of dielectrics are thus conducted on metal–insulator–semiconductor (MIS) structures. The interface state could be determinant for the dark current performances. Poor interface between dielectric and InP creates traps which generate parasitic carriers. The fixed charges in the dielectric can also have significant impact on the dark current by either creating a depletion region near the interface or accumulating charges and passivating this region.

**Figure 13:**
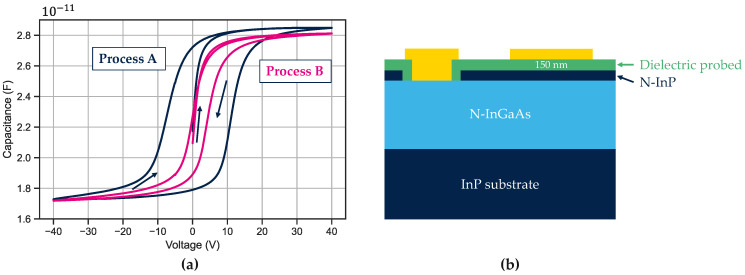
(**a**) Capacitance measurement on metal–insulator–semiconductor structure. The measurement starts at 0 V then ramps to +40 V then goes to −40 V and ends at +40 V. (**b**) A cross section of the MIS structure. The MIS is a 300 μm diameter circle.

A wide bias ramp of ±40 V is applied to the MIS structure (Figure 13b) while capacitance is measured. We use the Maserjian method to extract the flat band voltage (V_FB_) as explained in [36]. For each branch of the curve, we extract a V_FB_, and we compare the two extreme values. The delta is very different as we measured 18 V for process A and 4 V for process B (see Figure 13a). The V_FB_ hysteresis is directly proportional to the charge trapped in the dielectric as $Q_{traps}=-C_{dielectric}\;*\;\Delta V_{FB}$ [37]. Relatively, the passivation of process B traps less charges than process A.

**Figure 14:**
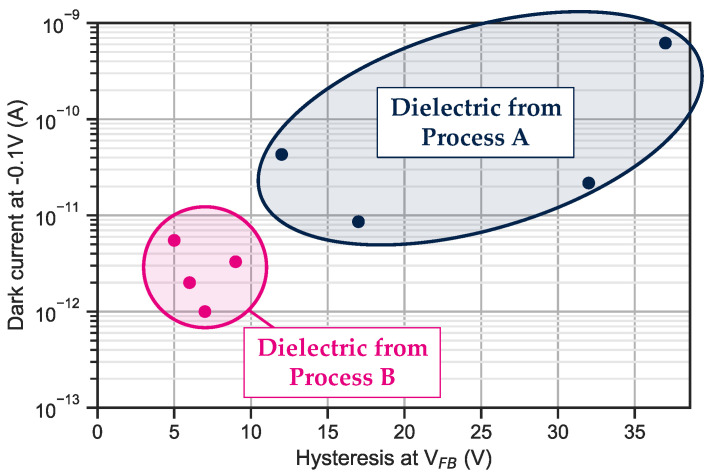
Dark current performances compared to the hysteresis measured on several different wafers.

This measurement protocol is conducted on various wafers. This hysteresis of the V_FB_ is plotted as a function of the dark current measured at −0.1 V and at room temperature for ten-by-ten 5 μm pixel pitch bundle. A correlation between V_FB_ hysteresis and I_dark_ is clearly visible in Figure 14.

As the dielectric properties could alter the device’s performances, it is thus very important to minimize the trap density at the InP/dielectric interface in the final device. Further investigations would allow quantification of the fixed charge and trap density for both processes A and B.

### 4.2. Photodiode Performances for 5 μm Pixel Pitch

To reach a pixel pitch as low as 5 μm for the photodiode fabrication, we use the passivation from process B which shows better dark current performances.

#### 4.2.1. Dark Current

The current noise limit of our set-up is estimated at 50 fA but the dark current from a single small pitch diode is below this value. Thus, we probe bundles of ten-by-ten diodes and the behaviour of one photodiode is extrapolated assuming all diodes behave homogeneously along the bundle.

**Figure 15:**
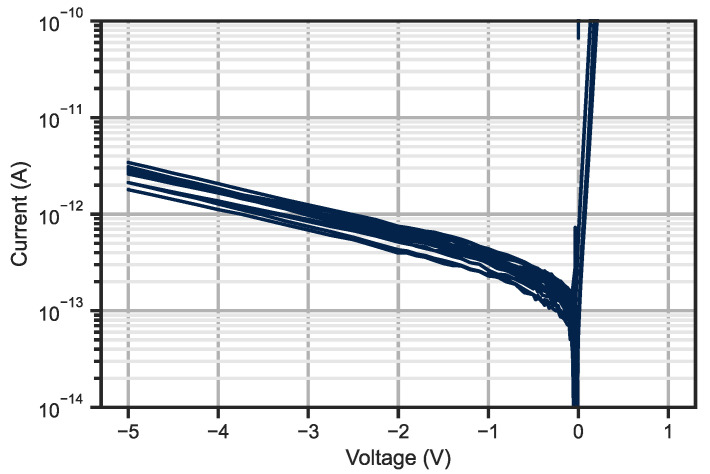
Dark current measurement of a ten-by-ten bundle of 5 μm pixel pitch photodiode. The measurements are conducted at 23 °C.

For dark current measurement, neighbouring photodiodes, as explained in the fabrication section, are biased with the same polarization as the measured diode to mimic an imager environment. Figure 15 shows the measured dark current of more than 10 bundles of photodiodes (i.e., 1000 photodiodes). The median dark current is 121 fA at −0.1 V which corresponds to a dark current density of 5 nA/cm^2^ at 23 °C. This dark current density is comparable to the state-of-the-art to date [3]. Previous results of the 5 μm pixel pitch photodiode presented in [38] were measured for a bundle of ten-by-ten bundle with unbiased neighbouring diodes.

For read-out integrated circuit concerns, we also measure the diode capacitance at −0.1 V and the typical value is 3 fF.

#### 4.2.2. Quantum Efficiency

The QE is an important figure of merit and quantifies the capacity of a photodiode to convert photons into collected charges (see Equation (2)):

(2) $$QE=\frac{number\;of\;collected\;charges}{number\;of\;photons\;sent}$$

**Figure 16:**
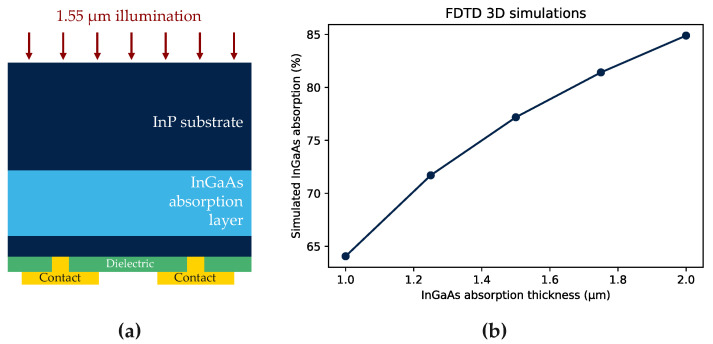
(**a**) Schematic test structure for QE measurement; (**b**) the results of the 3D FDTD simulations conducted with Lumerical to estimate the internal QE of the photodiode.

Finite-difference time-domain (FDTD) simulations are conducted with Lumerical software to estimate the theorical QE of the photodiodes. The structure has a 1.5 μm thick InGaAs layer. Based on the literature, the absorption coefficient considered here is $0.65\cdot 10^{4}\;{\mathrm{cm}}^{-1}$ [39]. Considering the layout and material configuration, the simulation predicts an absorption as high as 77.2% for a 1.5 μm InGaAs layer (see Figure 16b). This result estimates the internal QE as the reflection between air while InP is not considered in the simulation.

To measure the QE, we focus all the photons from a 10 μm diameter fibre in a 5 μm pitch ten-by-ten bundle (50 × 50 μm^2^ square). Photodiodes are illuminated from the back side, i.e., from the substrate, to avoid metallic layers in the optical path. The output power in Table 1 is measured with a power meter directly before entering the test structure. QE is measured for different laser powers to check the stability of the measurements.

The photocurrent is measured at −0.1 V for all output powers and the data are gathered in Table 1. The mean raw QE is about 54.1% and is constant with the rising optical power.

However, raw QE is lower than expected (see Figure 16) because photodiodes are measured with the InP substrate which impacts the photon transmission. The reflection between air and InP is 27% at 1.55 μm [40]. It is thus possible to extrapolate the QE considering the substrate removal and an ideal anti-reflective coating. In this case, the QE is estimated to reach 76.6%.

The mean expected QE is very similar to the one estimated by FDTD simulations. The value is consistent considering the experimental uncertainties such as alignment of the fibre in the optical system.

**Figure 17:**
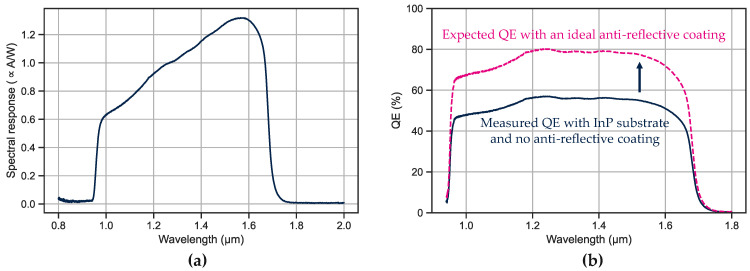
Measurements on the ten-by-ten bundle of photodiodes. (**a**) is the raw spectral response measured at −0.1 V and at room temperature; (**b**), QE in the SWIR range with the InP substrate (dark blue) and without the substrate and considering an ideal anti-reflective coating (dashed pink).

The spectral response of these photodiodes is then measured by Fourier transform infrared (FTIR) spectroscopy. The raw measurement is presented in Figure 17a. The spectral response is null for wavelengths lower than 0.93 μm because of the absorption in the InP substrate ($E_{G}$ = 1.34 eV, i.e., $\lambda_{c}$ = 0.93 μm).

To obtain the QE in the SWIR range, the spectral response is adjusted based on photon energy and a calibrated photodiode. The resulting QE curve in the SWIR range is shown in Figure 17b. It should be noted that the decrease in QE for wavelengths below 1.2 μm may be caused by free carrier absorption due to high *N*-type doping in the substrate. Further simulations are being conducted to determine the cause of this decrease.

Typical values of the quantum efficiency are gathered in Table 2 for material comparison.

#### 4.2.3. Sensor Spectral Noise under Illumination

**Figure 18:**
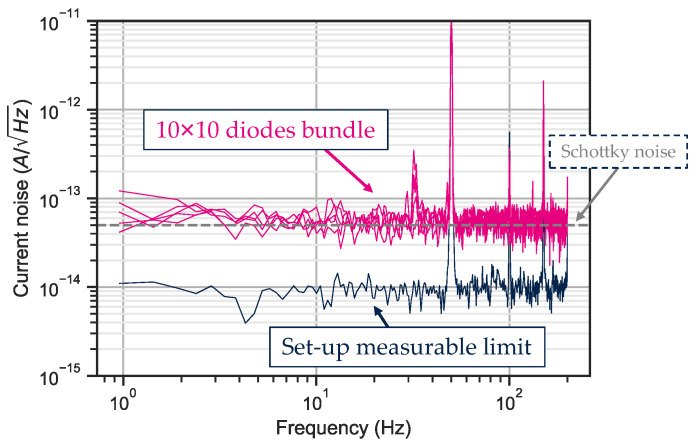
Current noise for a ten-by-ten 5 μm pixel pitch photodiode bundle measured at −0.1 V.

Current noise measurements were performed on several bundles under illumination. The test bench performs a time measurement of the current and the result is displayed in Figure 18 as a spectral current noise after applying a Fourier transform function.

The measured photodiode bundles on Figure 18 are limited by Schottky noise and do not show any 1/f trend noise at low frequency. This result confirms that the electrostatic barrier introduced by the InP cap layer along the collection path does not impact the carrier transport.

### 4.3. Towards Smaller Pixel Pitch: 3 μm Pixel Pitch Shallow-Mesa Photodiodes

To demonstrate that this innovative architecture is adapted for the fabrication of pixel pitch below 5 μm, we fabricated 3 μm pitch photodiodes.

**Figure 19:**
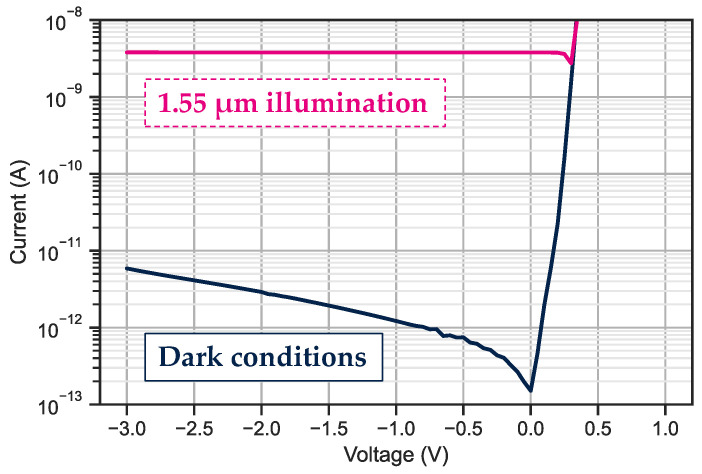
Median current measurement for bundles of one hundred 3 μm pixel pitch photodiodes under dark and SWIR illumination conditions. The dark blue line represents the dark current and the pink line is the photocurrent under 1.55 μm illumination.

The measurements of the dark current and photocurrent on bundles of ten-by-ten 3 μm pixel pitch photodiodes are presented in Figure 19. The collection of the photons is achieved at 0 V, indicating that there is no electrostatic barrier for the carriers generated in the InGaAs layer. The median dark current value is 0.28 pA at −0.1 V, which corresponds to a current density of 30 nA/cm^2^.

The successful 3 μm pixel pitch photodiode fabrication is demonstrated in Figure 19. The dark current density is higher than for larger pitches but the critical dimensions are close to our lithography tool limit. For such small pitch, actual process variations play an important role in the dark current performances. This architecture is promising for pitch reduction as we are now targeting adaptation of the process with up-to-date large format Si tools. The dark current density is constant with pixel pitch reduction from 15 μm to 5 μm indicating the absence of surface leakage between pixels. The measured dark current increases for 3 μm pixel pitch, probably due to lithography issues leading to electrical crosstalk between neighbouring pixels. In order to decrease the dark current, we are currently adapting the process to a CMOS compatible fab to access a more resolved scanner tool.

## 5. Discussion

**Figure 20:**
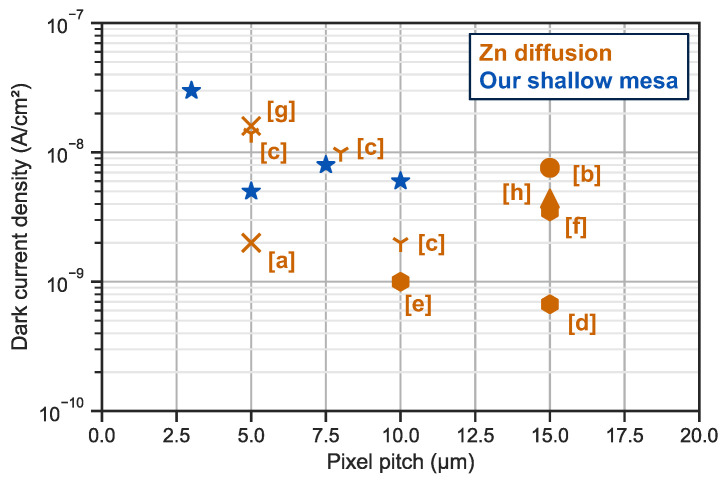
Comparison of our work in blue versus the state of the art for the fabrication of InGaAs photodiodes. Associated articles and measurement conditions if given: [a] 23 °C @−0.1 V article: [3]—[b] 30 °C @−0.2 V article: [4]—[c] 22 °C @−0.1 V article: [6]—[d] 20 °C article: [5]—[e] 20 °C article: [41]—[f] 30 °C article: [44]—[g] 25 °C article: [45]—[h] 25 °C @−0.3 V article: [15].

We benchmarked our architecture with state-of-the-art data of InGaAs photodiodes with small pitch and low dark current (see Figure 20) from the literature.

Our innovative structure shows major improvement compared to the mesa-type architecture published to date [18,26,43].

Zn diffused diodes in orange make up the standard industrial process for InGaAs photodiode fabrication and ref. [3] reports a dark current of 2 nA/cm^2^ for a 5 μm pixel pitch. From 10 to 5 μm pixel pitch, our diodes in blue show a flat dark current density of about 6 nA/cm^2^. The impact of charges in the dielectric or traps at the dielectric/cap interface could have a great impact on the dark current and is a clear way to improve the dark current density of these small pitch photodiodes.

The measurement for 3 μm pixel pitch is the first published for such a small pitch, and we obtain a dark current density as low as 30 nA/cm^2^. This slight increase in dark current density when the pitch reduces from 5 to 3 μm is under investigation and might be due to process imperfections as the critical dimensions are close to the tool limit.

As the pixel pitch reduces, crosstalk between pixels becomes a major concern. The typical diffusion length in such devices could reach tens of μm [46]. MFT measurements were performed on 15 and 10 μm pitches leading to suitable values [47]. Further investigations will be required to assess the impact of the crosstalk in our photodiodes with such small pitches.

## 6. Conclusions

With our innovative shallow mesa-type architecture, we demonstrated InGaAs photodiodes with promising results. We reached dark current as low as 5 nA/cm^2^ at −0.1 V and at room temperature for 5 μm pixel pitch. The measured external QE is 54% with the InP substrate and no anti-reflective coating. This value is expected to reach 76% with optimized anti-reflective coating and substrate removal. For lower pitch down to 3 μm, we successfully demonstrated the operation of photodiodes, even if the dark current density increases to 30 nA/cm^2^. Many improvement paths have been identified to reduce it. Future investigations with simulations and capacitive measurements will continue to quantify the impact of the charge in the dielectric and along the interface with the top layer. This promising architecture is now under study to be integrated as an imager with a Si-CMOS tools compatible process and hybridized on a Si-ROIC.

## Figures and Tables

**Table 1:** Measured QE and extrapolated QE with an anti-reflective coating.

Output Power (μW)	Photocurrent at −0.1 V (μA)	Raw QE (%)	QE with Ideal Anti-Reflective Coating (%)
86	55.9	53.5	75.7
97	61.3	53.2	75.3
107	69.6	55.2	78.1
117	78.1	54.6	77.2

**Table 2:** Typical values of quantum efficiency for the main absorption materials in SWIR range.

Absorption Material	Reference	External QE	Conditions
InGaAs	This work	77%	1.55 μm
InGaAs	[3]	>75%	1.2 μm
InGaAs	[41]	>80%	1.55 μm
Quantum dots	[42]	60%	1.4 μm
Ge on Si	[8]	52%	1.31 μm

## Data Availability

Data are contained in the article.
